# Disposition of Hexahydrocannabinol Epimers and Their Metabolites in Biological Matrices following a Single Administration of Smoked Hexahydrocannabinol: A Preliminary Study

**DOI:** 10.3390/ph17020249

**Published:** 2024-02-15

**Authors:** Annagiulia Di Trana, Alessandro Di Giorgi, Giorgia Sprega, Jeremy Carlier, Giorgi Kobidze, Eva Montanari, Omayema Taoussi, Giulia Bambagiotti, Maria Sofia Fede, Alfredo Fabrizio Lo Faro, Anastasio Tini, Francesco Paolo Busardò, Simona Pichini

**Affiliations:** 1 National Centre on Addiction and Doping, National Institute of Health, 00161 Rome, Italy; annagiulia.ditrana@iss.it; 2 Department of Biomedical Sciences and Public Health, University “Politecnica delle Marche”, 60126 Ancona, Italy; digiorgiale97@gmail.com (A.D.G.); giorgiasprega1996@gmail.com (G.S.); jerem.carlier@gmail.com (J.C.); kobidze.giorgi@yahoo.com (G.K.); eva.montanari@ospedaliriuniti.marche.it (E.M.); omayema.taoussi@gmail.com (O.T.); giuliabamba@gmail.com (G.B.); mariasofiafede13@gmail.com (M.S.F.); fabriziolofaro09@gmail.com (A.F.L.F.); anastasio.tini78@gmail.com (A.T.); f.p.busardo@staff.univpm.it (F.P.B.)

**Keywords:** hexahydrocannabinol epimers, pharmacokinetics, HHC metabolites, biological matrices

## Abstract

In 2023, hexahydrocannabinol (HHC) attracted the attention of international agencies due to its rapid spread in the illegal market. Although it was discovered in 1940, less is known about the pharmacology of its two naturally occurring epimers, 9(R)-HHC and 9(S)-HHC. Thus, we aimed to investigate the disposition of hexahydrocannabinol epimers and their metabolites in whole blood, urine and oral fluid following a single controlled administration of a 50:50 mixture of 9(R)-HHC and 9(S)-HHC smoked with tobacco. To this end, six non-user volunteers smoked 25 mg of the HHC mixture in 500 mg of tobacco. Blood and oral fluid were sampled at different time points up to 3 h after the intake, while urine was collected between 0 and 2 h and between 2 and 6 h. The samples were analyzed with a validated HPLC-MS/MS method to quantify 9(R)-HHC, 9(S)-HHC and eight metabolites. 9(R)-HHC showed the highest C_max_ and AUC_0–3h_ in all the investigated matrices, with an average concentration 3-fold higher than that of 9(S)-HHC. In oral fluid, no metabolites were detected, while they were observed as glucuronides in urine and blood, but with different profiles. Indeed, 11nor-9(R)-HHC was the most abundant metabolite in blood, while 8(R)OH-9(R) HHC was the most prevalent in urine. Interestingly, 11nor 9(S) COOH HHC was detected only in blood, whereas 8(S)OH-9(S) HHC was detected only in urine.

## 1. Introduction

Synthetic cannabinoids (SCRAs) stand as the primary category within New Psychoactive Substances (NPSs), encompassing over 250 variations as of 2023. Similar to other NPS groups, SCRAs emerged in the early 2000s as legal alternatives to the naturally occurring illegal cannabinoid Δ9-tetrahydrocannabinol (THC) [1,2]. These compounds originated from substances mimicking cannabinoids that alleviate pain, exhibiting a stronger affinity for the CB1 receptor compared to the CB2 receptors. Their widespread presence in the illegal market has raised substantial social and health concerns, associated with an increase in fatalities related to their use [3].

The European Monitoring Centre for Drugs and Drug Addiction (EMCDDA) reported a significant increase in seizures of a low-THC herbal cannabis material containing SCRAs, reaching 242 kg, which is 6.5 times higher than in 2020 and 1210 times higher than 2019 [1,2,4]. More recently, the subclass of semi-synthetic cannabinoids has raised concerns due to the growing popularity of new analogs like Δ8-THC and hexahydrocannabinol (HHC) [5,6]. Although HHC was discovered in 1940, its emergence in the United States’ drug market happened in late 2021, with its identification as a drug of abuse occurring in May 2022. Following the seizure of HHC-containing products in 20 EU Member States, the EMCDDA placed strict controls on HHC as a new NPS by March 2023. HHC can be easily synthesized from cannabidiol (CBD), extracted from low-THC cannabis [6].

**Figure 1:**
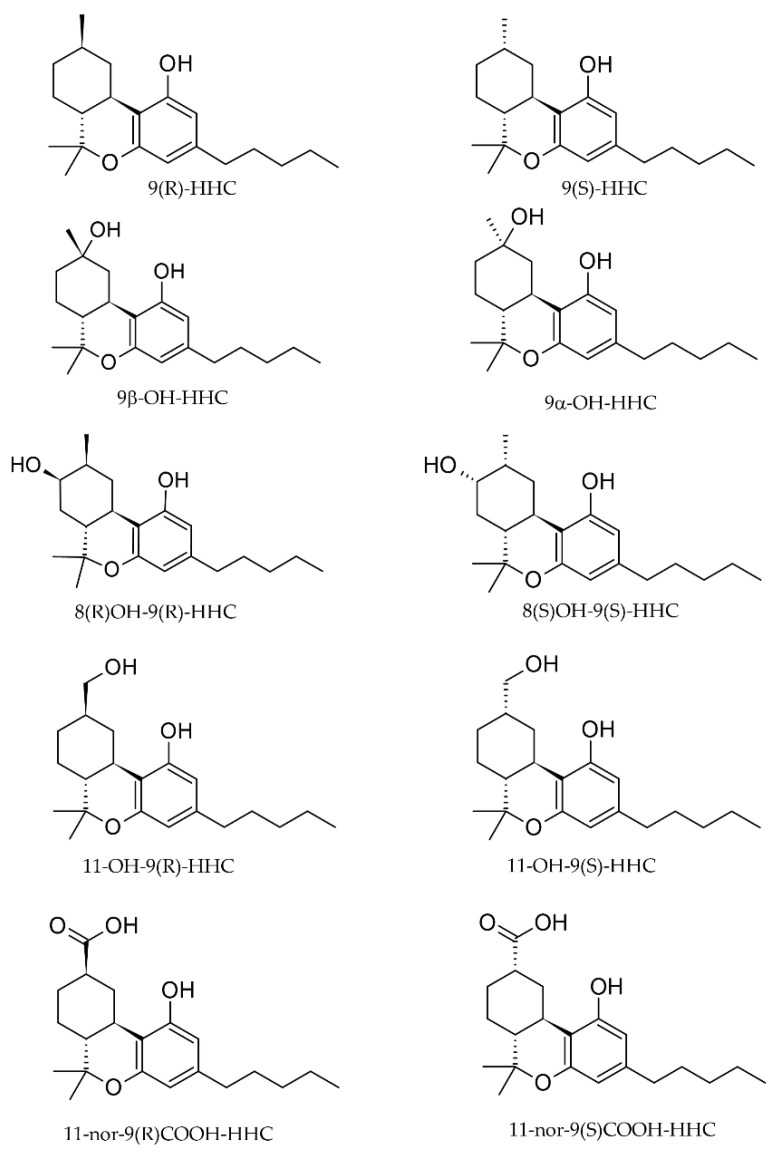
Chemical structures of hexahydrocannabinol epimers and the putative metabolites investigated in this study.

A hexahydro cyclohexyl ring structure characterizes HHC, which naturally occurs as two different epimers: 9(R)-HHC and 9(S)-HHC (Figure 1). According to preliminary studies, the epimers show different pharmacological activities. While 9(R)-HHC exhibits stronger “cannabis” effects, the 9(S)-epimer shows no activity even at high doses [5,6]. Furthermore, preliminary evidence suggested different pharmacokinetics for HHC epimers.

Recently, a few studies have described their metabolism in human hepatocytes in vitro and their metabolite excretion in urine, revealing important features about the different metabolic fates of the two epimers. However, most of the studies concern HHC mixed with other psychoactive substances such as THC or CBD [7,8,9,10]. Since 11-OH-HHC and 11nor-9-carboxy-HHC were demonstrated to be minor THC phase I metabolites, the co-consumption of other cannabinoids could interfere with the HHC epimers’ metabolism, resulting in a misleading metabolic pattern [9].

In this context, we sought to conduct a preliminary study, designing a naturalistic study on a limited cohort of healthy volunteers administered with a single controlled dose of a mixture 50:50 of 9(R)-HHC and 9(S)-HHC smoked with tobacco. Although HHC is commonly sold on the illegal market as a mixture with other cannabinoids, its administration without any other substance allowed us to investigate the metabolism of HHC and the disposition of its metabolites in whole blood, urine and oral fluid, excluding any interference in the metabolism.

## 2. Results

### 2.1. Subjects and Study Design

All recruited participants, four males and two women, completed the study, presenting the following characteristics: The male subjects had a mean age of 35.7 ± 7.8 years (range 26–45 years), mean weight of 81.7 ± 5.3 kg (range 75–87 kg) and a mean height of 182.0 ± 1.8 cm (range 180–184 cm). The two females presented a mean age of 27.0 ± 0.0 years (range 27 years), a mean weight of 69.5 ± 9.5 kg (range 60–79 kg) and a mean height of 167.7 ± 1.1 cm (range 167–169 cm). Although all the subjects had a history of occasional use of cannabis and/or light cannabis use at least once in their life, none of them is a regular consumer of cannabinoids, as confirmed by the toxicological screening performed before the trial. Regarding smoking habits and alcohol consumption, all the participants are social consumers of alcohol, while 50% of the participants regularly smoke tobacco, even in association with alcohol consumption.

The subjects were asked to smoke a whole cigarette, manually rolled with 500 mg of tobacco and 25 mg of HHC pure extract, composed of 9(R)-HHC:9(S)-HHC, 50:50 *w*/*w*, as confirmed by the analysis performed before the trial. All the biological samples were collected after the subjects finished the cigarette. We studied the disposition of HHC epimers in blood, urine and oral fluid, while the metabolites were not detected in the latter matrix. However, the disposition of HHC metabolites and of the parent drug epimers were assessed in blood and urine, evaluating the accumulation in 6 h urine.

### 2.2. HHC Epimers Concentration–Time Profile and Pharmacokinetics in Blood

**Figure 2:**
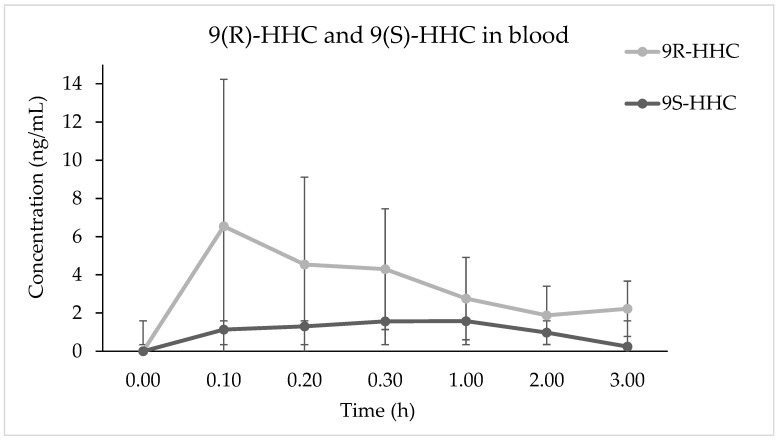
Time course of 9(R)-HHC and 9(S)-HHC median concentrations (*n* = 6, median values ± standard error) in blood following the smoking of 25 mg of a mixture of 9(R)-HHC:9(S)-HHC 50:50 *w*/*w* in 500 mg tobacco.

The time course of median (*n* = 6, ± SE) concentrations of 9(R)-HHC and 9(S)-HHC in blood, up to 3 h after administration, is represented in Figure 2. Notably, the median concentration is higher for 9(R)-HHC than for 9(S)-HHC at every time point, with the peak concentration of 9(R)-HHC 3-fold higher than that of 9(S)-HHC (Table 1). As a consequence, the area under the concentration–time curve (AUC_0–3h_) differs for the epimers, and is higher for 9(R)-HHC. While the peak time is comparable, 9(S)-HHC is more rapidly eliminated with an average (*n* = 6) apparent half-life of 1.3 (±10.8, SD) hours and an elimination constant of 0.2 (±0.3). However, the calculated clearance is 2-fold higher for 9(S)-HHC than 9(R)-HHC.

### 2.3. HHC Metabolites Concentration–Time Profile and Pharmacokinetics in Blood

**Figure 3:**
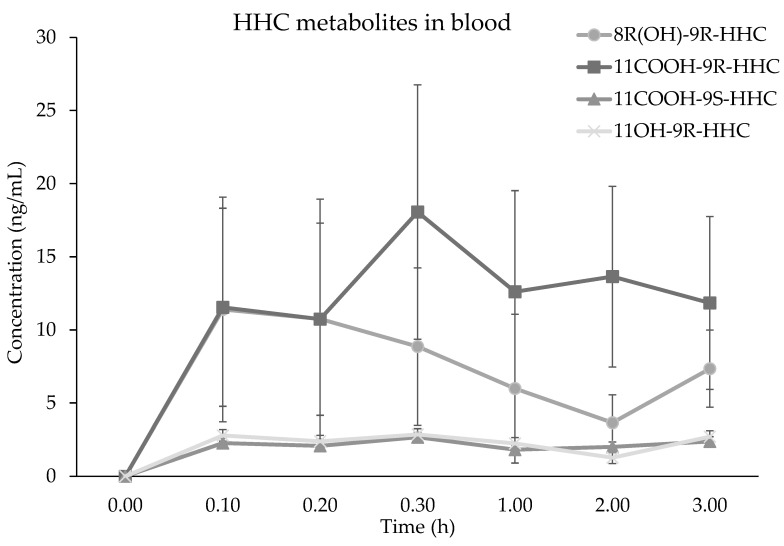
Time course of 8(R)OH-9(R) HHC, 11OH-9(R)-HHC, 11nor-9(R) COOH HHC and 11nor-9(R) COOH HHC median concentrations (*n* = 6, median values ± standard error) in blood following the smoking of 25 mg of a mixture of 9(R)-HHC:9(S)-HHC 50:50 *w*/*w* in 500 mg tobacco.

The metabolites detected in the blood samples were 8(R)OH-9(R) HHC, 11OH-9(R)-HHC, 11nor-9(R) COOH HHC and 11nor-9(S) COOH HHC, present as glucuronic acid conjugates. The metabolites’ median (*n* = 6, ± SD) concentrations in blood up to 3 h after the administration are presented in Figure 3. Notably, only the carboxy metabolites were both in R and S configurations, with the concentration of 11nor-9(R) COOH HHC C_max_ 7-fold higher than that of 11nor-9(S) COOH HHC (Table 1). Furthermore, 11nor-9(R) COOH HHC was the most prevalent metabolite at every time point, with a later peak time (1.2 ± 1.35 h) and a higher bioavailability (AUC_0–3h_ 43.7 ± 48.2). Its metabolic precursor 11OH-9(R)-HHC was detected in low concentrations at every time point, with very slow elimination due to its K_e_ = 0.01 (±0.4, SD). The main hydroxylated metabolite was 8(R)OH-9(R)-HHC. It shows a high C_max_ and a rapid onset (Table 1). However, it is rapidly eliminated with a half-life of 1.5 h.

### 2.4. HHC Concentration–Time Profile and Pharmacokinetic in Oral Fluid

**Figure 4:**
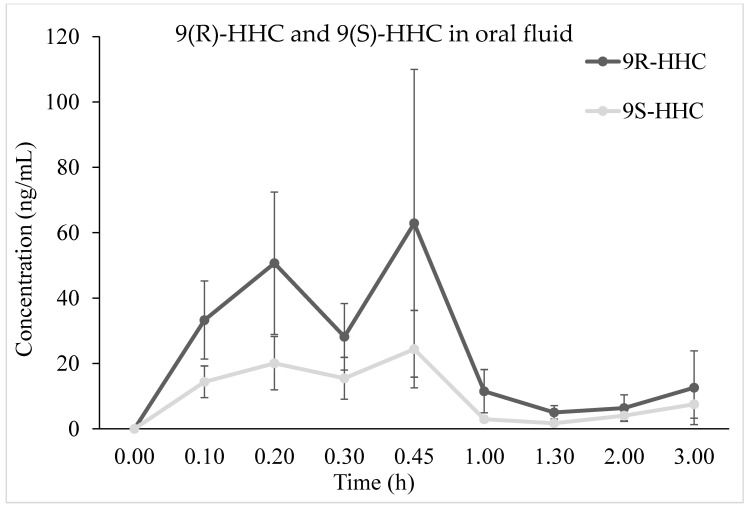
Time course of 9(R)-HHC and 9(S)-HHC median concentrations (*n* = 6, mean values ± standard error) in oral fluid following the smoking of 25 mg of a mixture of 9(R)-HHC:9(S)-HHC 50:50 *w*/*w* in 500 mg tobacco.

The time course of 9(R)-HHC and 9(S)-HHC mean concentrations (*n* = 6) in oral fluid up to 3 h after the administration is presented in Figure 4. Similarly to that in blood, the concentration of 9(R)-HHC is about 3-fold higher of that of 9(S)-HHC in all the samples, with a C_max_ of 100.6 and 35.1, respectively. The concentration–time courses are mostly comparable between 9(R)-HHC and 9(S)-HHC, whereas the concentration of 9(R)-HHC is mostly above 9(S)-HHC. Consequently, the concentration of 9(R)-HHC AUC_0–3h_ is greater than that of 9(S)-HHC. However, the two epimers’ peak times are comparable (Table 2). Moreover, the elimination constant is similar for 9(R)-HHC and 9(S)-HHC (Table 2), although the clearance suggests that 9(S)-HHC is more rapidly eliminated. Finally, both the epimers have the same apparent half-life.

### 2.5. Urinary Excretion of HHC Epimers and Their Metabolites following the Administration of 25 mg Smoked HHC Epimers

**Figure 5:**
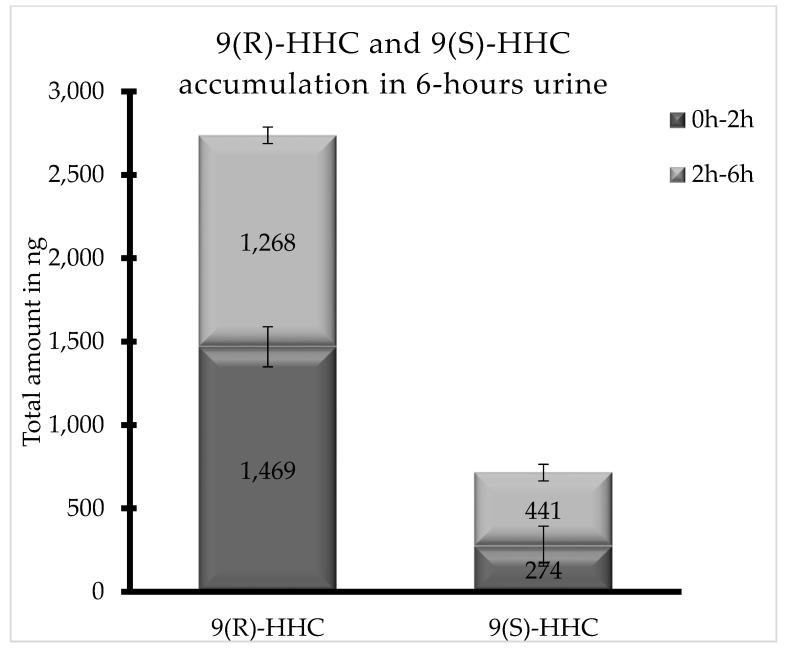
Total accumulation (ng) of 9(R)-HHC and 9(S)-HHC glucuronic acid conjugates between 0 and 2 h and between 2 and 6 h following the smoking of 25 mg of a 9(R)-HHC:9(S)-HHC 50:50 *w*/*w* mixture with 500 mg tobacco.

Both HHC epimers were excreted in up to 12 h urine as glucuronic acid conjugates. As shown in Figure 5, 9(R)-HHC was by far the most excreted HHC epimer in 6 h urine (2737 ng total), with a similar accumulation between 0 and 2 h, whereas the total excreted amount of 9(S)-HHC in 6 h urine is 715 ng, with a significant variation between the two considered time ranges.

**Figure 6:**
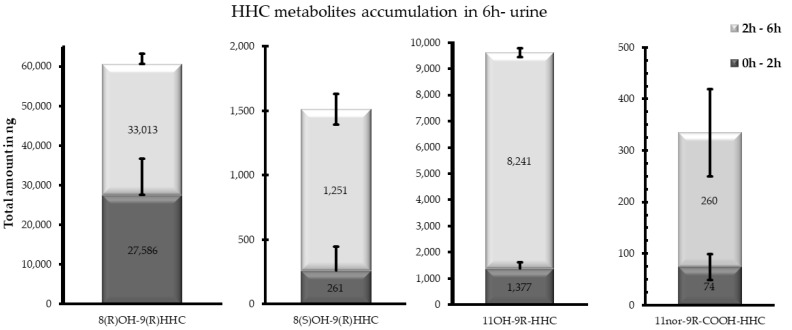
Total accumulation (ng) of 8(R)OH-9(R) HHC, 8(S)OH-9(S)-HHC, 11OH-9(R)-HHC and 11nor-9(R) COOH HHC between 0 and 2 h and between 2 and 6 h following the smoking of 25 mg of a 9(R)-HHC:9(S)-HHC 50:50 *w*/*w* mixture with 500 mg tobacco.

Differently from the other investigated matrices, the metabolites detected were 8(R)OH-9(R) HHC, 8(S)OH-9(S)-HHC, 11OH-9(R)-HHC and 11nor-9(R) COOH HHC, excreted as conjugated metabolites. Indeed, 11nor-COOH-9(S)-HHC was not detected in any sample (Figure 1). Interestingly, all the metabolites were excreted in different amounts between the considered time ranges, with a considerable accumulation between 2 and 6 h (Figure 6). Furthermore, the carboxy metabolite was the least accumulated metabolite in both the time ranges, with a total accumulation of 334 ng in 6 h.

Considering both the time ranges, the main metabolite was 8(R)OH-9(R) HHC, with a total accumulation of 60,599 ng, with a comparable elimination between 0 and 2 h and 2 and 6 h. In comparison, the 8(S)OH-9(S) HHC epimer was represented by a negligible quantity (total accumulation 1512 ng), and was prevalently eliminated between 2 and 6 h. Finally, the total accumulation of 11OH-9(R)-HHC in urine amounted to 9618 ng (Figure 5).

## 3. Discussion

To date, only a few studies have investigated the pharmacological profile of HHC and its disposition in biological matrices, as this new semisynthetic cannabinoid has only recently emerged on the illicit market [7,8,9,10]. The analysis of urine samples from two different HHC users allowed us to detect several metabolites of both 9(R)-HHC and 9(S)-HHC. In this study, HHC epimers and their phase I metabolites were all excreted as glucuronides, while the most intense chromatographic signal corresponded to the 11-OH-HHC metabolite [7]. Furthermore, the different routes of administration resulted in different metabolite dispositions in urine, as already observed for THC and CBD [11,12,13].

In this study, a mixture of 50:50 *w*/*w* 9(R)-HHC and 9(S)-HHC was administered to the subjects, without any other psychoactive substance or cannabinoid such as THC or CBD, to assess the disposition of the two drugs without any possible interference. According to the limited available data, the administered dose is sufficient to exert a psychotropic effect without any relevant adverse effects. Moreover, the analytical discrimination between 9(R)-HHC and 9(S)-HHC and their epimeric metabolites allowed us to consider the possible differences in the pharmacokinetic profile, since 9(R)-HHC and 9(S)-HHC showed different pharmacological potency in animal models [5,6]. In this regard, 9(R)-HHC showed a greater bioavailability and a higher peak concentration, with a slower elimination, while both the epimers presented a rapid onset in blood with a similar T_max_. Indeed, the pharmacokinetics of 9(R)-HHC may contribute to the higher pharmacological potency of the epimers, as previously reported in animal models [5,6]. As already described for urine samples, the investigated metabolites were detected only as glucuronides both in urine and blood, while metabolites were not detected in OF [7].

As expected, HHC epimers showed a similar profile in oral fluid and blood. Indeed, 9(R)-HHC and 9(S)-HHC reached a maximum concentration above 100 ng/mL in about 30 min and showed similar elimination. As a consequence, oral fluid appeared as a promising alternative biological matrix for monitoring and toxicological analysis, as already observed for other phytocannabinoids and synthetic cannabinoids [11,12,14]. Furthermore, the significant elimination of 9(R)-HHC and 9(S)-HHC may play a role in the observed concentration in blood, which appears lower than that of the corresponding metabolites since the first considered time point. However, the hypothesis should be corroborated a larger study in which other parameters could be investigated.

Differences in the disposition of the investigated epimers were also observed in urine, where the excreted amount of 9(S)-HHC in 6 h was almost double than that of 9(R)-HHC, although the administered dose was the same. A stereoselective mechanism of elimination may be hypothesized due to the stereoselectivity of glucuronidation, as already described for a number of other drugs [15,16].

More complex is the role of metabolic enzyme enantioselectivity when two subsequent metabolic reactions occur, such as glucuronidation following oxidation for 11OH-9(R)-HHC. In fact, the corresponding epimers were not detected in any of the investigated matrices, suggesting that 9(S)-HHC is prevalently oxidated on carbon 9, while position 11 is more prone to oxidation in the case of 9(R)-HHC. Indeed, the hydroxylation on position 11 was also observed in the metabolic patterns of Δ9-THC and Δ8-THC, producing 11OH-Δ9-THC and 11OH-Δ8-THC, respectively, but it is considered a minor metabolite. Conversely, 11OH-9(R)-HHC was the second most excreted metabolite in urine, with a remarkable accumulation between 2 and 6 h.

Differently from other cannabinoids, hydroxylation in position 8 was observed for both the epimers, appearing as the most preferred hydroxylated metabolite. However, it is surprising that only 8(R)OH-9(R)-HHC was detected in blood and urine, while 8(S)OH-9(S)HH was detected only in urine. In this regard, the last one was mostly accumulated between 2 and 6 h, with a negligible accumulation in the first time range. This could suggest a slower formation of 8(S)OH-9(S)HHC than the other metabolites. Furthermore, the ratio between the carboxy metabolite and the hydroxy metabolite is inverted between urine and blood, with 11nor-9(R)-COOH-HHC being the main metabolite.

In this regard, the possible biomarker for the two epimers differs from matrix to matrix. Indeed, 8(R)OH-9(R)-HHC appears to be a good long-term urinary biomarker to prove 9(R)-HHC consumption, while 11nor-9(R) COOH HHC proved to be the most suitable biomarker in blood. Similarly, 8(S)OH-9(S)-HHC and 11nor-9(S) COOH HHC could be good 9(S)-HHC biomarkers in urine and blood, respectively.

Furthermore, the lack of studies on metabolism and the unavailability of analytical standards might have limited the detection of other metabolites. Finally, the volunteers were prevalently males, preventing the evaluation of possible differences between genders.

### Study Limitations

All the presented data should be considered as preliminary results due to the weaknesses of the study: the limited extension of the follow-up, the restricted number of healthy volunteers, the relatively low dose administered and the unique considered route of administration. Therefore, the reported parameters should be confirmed by a comprehensive experimental study considering a larger number of subjects observed for a prolonged time.

## 4. Materials and Method

### 4.1. Subjects Enrolment

Six subjects, four men and two women, were initially recruited. Eligibility criteria included successful accomplishment of a general physical examination, routine laboratory analysis and electrocardiogram. The exclusion criteria were positivity to cannabinoids at a drug screening test and prior HHC intake. All subjects gave written informed consent, and the study was conducted in accordance with the Declaration of Helsinki and approved by the Institutional Ethics Committee (IRCCS-INRCA Ancona). Subjects participated as outpatients in a 6 h experimental session in the Ancona Institute of Patient Care and Scientific Research (IRCCS-INRCA) room equipped for clinical trials with an open balcony where they could smoke ad libitum.

### 4.2. HHC Cigarettes Preparation

A resinous HHC extract was purchased on the internet as “light cannabis” product before the substance was scheduled under the national law on drugs of abuse. Before the trial, the extract was analyzed in LC-MS/HRMS and HPLC-MS/MS to assess the purity and quantify the epimer contents (50:50 *w*/*w* of 9(R)HHC and 9(S)HHC). Seven different cigarettes were prepared by mixing 25 mg of the extract with 500 mg of ground blond tobacco, rolled just before consumption.

### 4.3. Study Design

The study was naturalistic, not randomized, and single-blinded since the subjects were informed only on the cannabinoid preparation but not of the administered dose. After an overnight fast, the subjects were admitted to the University Politecnica delle Marche (UNIVPM) at 08:00 a.m. in a furnished room with free access to a balcony, where they remained for the entire experimental session under observation. Upon arrival, the subjects signed an informed consent form and declared to have not consumed psychoactive drugs for at least seven days before the study. The last cigarette allowed was 2 h before starting the session, with smoking being prohibited during the study. Samples of blood, urine and oral fluid were collected before the administration (baseline, 0 h) to assess the presence of any substance that might have interfered with the study. The enrolled participants were requested to smoke one cigarette each ad libitum and were assisted for 6 h at the university.

Blood samples were collected at 10 min, 20 min, 30 min, 1 h, 2 h and 3 h after smoking a single cigarette. Similarly, OF samples were collected before and 10, 20, 30 and 45 min, 1, 1.5, 2 and 3 h after smoking. Furthermore, pooled urine samples were collected between 0–2 h and 2–6 h.

### 4.4. Quantification of HHC Epimers and Their Metabolites in Blood, Oral Fluid and Urine Samples

A previously developed and fully validated HPLC-MS/MS (1290 Infinity II coupled to a 6470A Triple Quadrupole, Agilent, CA, USA) method for the simultaneous quantification of 9(R)- HHC and 9(S)-HHC was carried out [17]. For OF and blood, 100 μL of each was spiked with 10 μL of internal standard (THCd_3_) and was liquid–liquid extracted with n-hexane/ethyl acetate 9:1 (*v*/*v*) with 10% of acetic acid. Before the liquid–liquid extraction of 100 μL urine samples, chemical hydrolysis with NaOH 5 M at 70 °C for 30 min was conducted to deglucuronise all the metabolites. The analytes were chromatographically separated through an immobilized amylose tris(3-chloro-5-methylphenylcarbamate)-based chiral column Lux i-Amylose-3 (Phenomenex, CA, USA) in isocratic elution mode with methanol/water at a flow rate of 0.5 mL/min. The analytes were ionized with an electrospray ionization source operating in positive mode. 

The same extraction protocol was applied for 9β-OH-HHC, 9α-OH-HHC, 8(S)OH-9(S)-HHC, 8(R)OH-9(R)-HHC, 11-OH-9(S)-HHC, 11-OH-9(R)-HHC, 11-nor-9(S)COOH-HHC and 11-nor-9(R)COOH-HHC, which were separated through a Lux AMP (Phenomenex, CA, USA) chiral column with methanol and water at 80/20 (*v*/*v*) in isocratic elution. All the metabolites were ionized by an ESI source operating in negative mode. Two different mass spectrometric transitions were selected for each analyte: a qualitative and a quantitative transition. The same instrumental conditions were applied to quantify 9(R)-HHC and 9(S)-HHC in the extract.

An in-house qualitative screening method using an LC-HRMS/MS (Dionex UltiMate 3000 chromatographic system coupled with a Thermo Scientific (Waltham, MA, USA) Q Exactive was applied to assess the presence of other psychotropic substances in HHC light cannabis extract and in the biological samples collected before the administration of HHC [18].

### 4.5. Statistical Analysis

The maximum concentration (C_max_), the time needed to reach maximum concentrations (T_max_), AUC_0–3h_, the elimination half-life (t_1/2_) and the elimination constant (K_e_, calculated with at least three sample points) for HHC epimers and their metabolites in blood and OF were determined using Pharmacokinetic Functions for Microsoft Excel (Joel Usansky, Atul Desai, and Diane Tang-Liu, Department of Pharmacokinetics and Drug Metabolism, Allergan, Irvine, CA, USA, at https://www.coursehero.com/file/30859156/pkfdoc/ (Accessed: 20 June 2023). The parameters were calculated for each subject, considering the quantified concentration of each analyte at each considered time point, and the average was calculated and is reported in Table 1 and Table 2.

## 5. Conclusions

The recent emergence of the semisynthetic cannabinoid HHC has attracted particular attention from national and international institutions due to its potential spread as an illicit substance. Although this molecule was first synthesized in the 1940s, little information on its pharmacokinetic profile is currently available, representing a challenge for health personnel and toxicologists. Since some evidence suggests different pharmacological profiles of the epimers of HHC, it is important to study their disposition in the principal biological matrices in order to elucidate the possible different fates of the two substances. In this naturalistic study, we investigated the pharmacokinetics of an equal dose of 9(R)-HHC and 9(S)-HHC, smoked with 500 mg of tobacco, in six healthy volunteers. As expected, the stereochemistry of the compounds influenced the pharmacokinetics of the epimers. Indeed, 9(R)-HHC presented a higher bioavailability and a slower elimination. Furthermore, the metabolic fate differed for 9(R)-HHC and 9(S)-HHC, with 8(R)OH-9(R)HHC and 11OH-9(R)-HHC being the most accumulated metabolites in urine, while 11nor-9(R) COOH HHC was the main metabolite in blood. Finally, OF was demonstrated to be a suitable matrix for HHC consumption screening, being detectable up to 3 h after consumption for both epimers. Despite the internal limitations of this study, the obtained results revealed notable features that should be considered for further investigations. More comprehensive studies with a wider cohort of volunteers are needed to clarify the pharmacokinetics of HHC epimers and assess the possible influence of other factors such as the interference of smoked tobacco on the absorption and metabolism of HHC epimers. Furthermore, other administration routes should be considered.

## Figures and Tables

**Table 1:** Pharmacokinetic parameters of HHC epimers and their metabolites in the blood of healthy volunteers following the smoking of 25 mg of a 9(R)-HHC:9(S)-HHC 50:50 *w*/*w* mixture with 500 mg tobacco.

Pharmacokinetics Parameters (Mean ± SD)						
Analytes	C_max_ (ng/mL)	T_max_ (h)	AUC_0–3h_ (ng/mL∗h)	K_e_	T_1/2_	Cl
9(R)-HHC	7.9 ± 7.3	0.3 ± 0.3	7.6 ± 5.2	0.2 ± 0.3	1.3 ± 10.8	4.8 ± 4.6
9(S)-HHC	2.3 ± 1.3	0.3 ±0.7	3.3 ±1.4	0.8 ± 0.7	1.6 ± 2.0	10.0 ± 7.7
11COOH-9(R)-HHC	25.9 ± 20.3	1.2 ± 1.35	43.7 ± 48.2	0.2 ± 0.5	0.8 ± 2.5	4.7 ± 9.1
11COOH-9(S)-HHC	3.4 ± 3.9	0.3 ± 0.7	6.9 ± 9.1	0.0 ± 0.2	1.4 ± 3.1	10.3 ± 7.4
11OH-9(R)-HHC	4.3 ± 3.3	0.3 ± 0.3	6.2 ± 4.3	0.01 ± 0.4	10.4 ± 22.9	4.5 ± 4.1
8(R)OH-9(R)-HHC	14.9 ± 19.0	0.2 ± 1.6	18.2 ± 23.8	0.1 ± 0.4	1.5 ± 3.2	2.9 ± 3.8

Abbreviations: AUC_0–3h,_ area under the concentration–time curve at 3 h; Cl, clearance; C_max_, maximum concentration; Ke, elimination constant; SD, standard deviation; T_max_, time to reach maximum concentration (hour); T_1/2_, apparent half-life.

**Table 2:** Pharmacokinetic parameters of HHC epimers and their metabolites in oral fluid of healthy volunteers following the smoking of 25 mg of a 9(R)-HHC:9(S)-HHC 50:50 *w*/*w* mixture with 500 mg tobacco.

Pharmacokinetics Parameters (Mean ± SD)						
Analytes	C_max_ (ng/mL)	T_max_ (h)	AUC_0–3h_ (ng/mL∗h)	K_e_	T_1/2_	Cl
9(R)-HHC	100.6 ± 96.5	0.2 ± 0.3	1.9 ± 1.2	32.5 ± 19.1	0.3 ± 0.2	24.6 ± 22.9
9(S)-HHC	35.1 ± 23.9	0.3 ± 0.1	0.7 ± 0.5	8.4 ± 12.4	0.2 ± 0.8	43.7 ± 28.0

Abbreviations: AUC_0–3h_, area under the concentration–time curve at 3 h; Cl, clearance; C_max_, maximum concentration); K_e_, elimination constant; SD, standard deviation; T_max_, time to reach maximum concentration (hour); T_1/2_, apparent half-life.

## Data Availability

Data sharing is not applicable.
